# Screen-Free Time With Friends to Promote Face-to-Face Peer Interaction and Reduce Recreational Screen Use Among Children Aged 9-11 Years: Protocol for a Cluster Randomized Controlled Trial

**DOI:** 10.2196/84831

**Published:** 2026-06-05

**Authors:** Sara Kruse Lange, Sarah Overgaard Sørensen, Teresa Victoria Høy, Kristian Traberg Larsen, Anders Blædel Gottlieb Hansen, Russell Jago, Peter Lund Kristensen, Anders Grøntved, Mette Toftager, Anne Kær Gejl

**Affiliations:** 1 Department of Sports Science and Clinical Biomechanics, Faculty of Health Sciences, University of Southern Denmark, Campusvej 55, Odense, 5230, Denmark, 45 65507908; 2 National Institute of Public Health, Faculty of Health Sciences, University of Southern Denmark, Copenhagen, Denmark; 3 Center for Clinical Research and Prevention, Copenhagen University Hospital, Frederiksberg, Denmark; 4 Population Health Sciences, Bristol Medical School, University of Bristol, Bristol, United Kingdom

**Keywords:** social interaction, interpersonal relations, digital media use, screen time, physical activity, mental health, well-being, child, community participation, after-school programs

## Abstract

**Background:**

Children’s leisure time has changed in recent decades—with more time spent on screen media and less time face-to-face with peers—potentially affecting their physical and mental well-being.

**Objective:**

This protocol outlines a cluster randomized controlled trial evaluating the Screen-Free Time With Friends intervention, which aims to promote face-to-face peer interaction and reduce recreational screen media use among children aged 9‐11 years.

**Methods:**

The study is conducted as a cluster randomized controlled trial involving 9-11-year-old children and their parents from 18 schools in Denmark. Schools are randomized (1:1) to either a control or an intervention group. The intervention includes five core components: (1) curricular activities, (2) two family meetings, (3) a brief parent exercise, (4) a development program for after-school clubs, and (5) two community workshops. It is designed to allow local adaptation while maintaining fidelity to the core components. Primary and key secondary outcomes, including face-to-face peer interaction across leisure domains and solitary screen time, are assessed at baseline, 6‐10 months, and 13‐15 months follow-up. Additional exploratory outcomes (e.g., leisure activities, social relations, and well-being) are measured at baseline and follow-up. Time spent in face-to-face interactions with peers was assessed using a short SMS-based questionnaire sent to registered parents. The instrument has not been formally validated but was tested in a feasibility study. A comprehensive process evaluation explores implementation, context, and mechanisms of change.

**Results:**

The project was funded in July 2020. Schools were recruited from July 2023 to January 2024, while children and parents were recruited from November 2023 to April 2024. At baseline, 685 children were enrolled at the participating schools, with 343 (50%) having at least one parent enrolled in the questionnaire. The primary outcome is therefore assessed among a subset of participants, which may affect the effective sample size. Baseline data were collected from December 2023 to June 2024, and follow-up data from March to August 2025. As of March 2026, data analysis has not yet commenced, and no study results are currently available. Findings are expected to be published by the end of 2026.

**Conclusions:**

Regardless of the findings, this study will generate important knowledge about the intervention’s potential effectiveness, complemented by insights into its implementation, context, and mechanisms of change. These insights can assist municipalities, schools, after-school clubs, community stakeholders, and parents in shaping everyday environments that foster children’s face-to-face interaction and encourage balanced screen use. The findings may inform policy decisions, guide the development of new national and local initiatives, and inspire future research into feasible, real-world interventions that support meaningful and enriching leisure experiences for children.

## Introduction

In modern society, the use of screen media among children and adolescents has increased rapidly in recent years [1]. Although digital technologies offer several advantages, including enhanced communication and educational opportunities, increasing evidence points to the potential negative impacts of screen media use among children and adolescents [2-4]. Excessive screen use has been associated with a range of adverse physical and mental health outcomes, including obesity, emotional and cognitive difficulties, reduced well-being, and elevated risk of lifestyle-related health issues [2-4]. Potential mechanisms include both direct time displacement and changes in social preferences, opportunities, and norms. Time is a finite resource, and time spent on one activity may reduce the time available for others. Empirical studies provide support for the displacement of physical activity and sleep by screen-based activities [3,5,6], whereas evidence for the displacement of face-to-face social interaction is more inconsistent [7,8]. However, at the population level, a shift in time use has been observed, with time spent in face-to-face interaction with peers declining markedly in Denmark alongside increases in screen-based media use. In 2009, 72% and 71% of Danish 11-year-old boys and girls, respectively, spent time face-to-face with friends more than once a week [9]. In 2021, the proportion had decreased to 40% of boys and 60% of girls [9]. Another Danish study demonstrated that the proportion of 11-year-old children spending time after school with friends 4 to 5 days per week has decreased from 54% in 1988 to 19% in 2022 for girls and from 58% to 24% for boys [10]. Similar declines in face-to-face social interaction with peers have also been reported internationally among adolescents (aged 13‐18 years) in the United States [7].

Face-to-face interaction and play are essential for children’s development, fostering social and emotional skills, creativity, and problem-solving abilities [11]. In contrast, time spent alone has been associated with increased risk of anxiety, depression, poor friendship quality, and school difficulties [12]. Moreover, the decline in peer face-to-face interaction—alongside a rise in recreational screen use—may further reflect mutually reinforcing behaviors [13-15] that together contribute to the low levels of physical activity observed among Danish children, as shown in a recent Danish report based on accelerometer measurements [16]. Collectively, these trends pose a serious risk to both the physical and mental well-being of children.

Consequently, there is growing concern about the amount of time children spend on recreational, solitary screen use and how this may affect their physical and mental well-being. In response, the Screen-Free Time With Friends intervention was developed to explore whether promoting face-to-face interaction as an alternative to recreational screen use can influence key aspects of children’s leisure-time behavior and well-being.

This cluster randomized controlled trial (RCT) examines the effectiveness of the Screen-Free Time With Friends intervention among children aged 9‐11 years. A process evaluation is conducted alongside the implementation of the intervention. This protocol outlines the methodology and procedures for conducting the cluster RCT in accordance with the SPIRIT (Standard Protocol Items: Recommendations for Interventional Trials) 2013 checklist and is registered in clinicaltrials.gov (NCT06163495).

The objectives of this study are as follows:

1. *Time spent face-to-face with peers:* To evaluate the effectiveness of the Screen-Free Time With Friends intervention in increasing children’s time spent face-to-face with peers across different leisure time domains.
2. *Time spent face-to-face with peers in different leisure time domains:* To examine the effectiveness of the Screen-Free Time With Friends intervention in increasing children’s time spent face-to-face with peers in different leisure time domains, including after-school clubs, organized leisure activities, and unorganized leisure activities.
3. *Solitary screen time:* To evaluate the effectiveness of the Screen-Free Time With Friends intervention in reducing children's time spent using screen media alone during leisure time.

Based on the study objectives, the following hypotheses will be examined:

1. Children in intervention schools will show a greater increase in time spent face-to-face with peers from baseline to follow-up compared with children in control schools.
2. Children in intervention schools will show greater increases from baseline to follow-up in time spent face-to-face with peers in specific leisure time domains compared with children in control schools:
  - in after-school clubs,
  - in organized leisure activities, and
  - in unorganized leisure activities.
3. Children in intervention schools will show a greater reduction from baseline to follow-up in solitary screen use compared with children in control schools.

The study also includes several exploratory outcomes that will further inform the potential effectiveness of the intervention and support hypothesis generation for future research. These outcomes include measures of children’s leisure activities, social relationships, and well-being.

## Methods

### Feasibility Trial

From February to November 2023, we conducted the Screen-Free Time With Friends feasibility trial including 3 schools, the related after-school clubs, and the local communities from 3 different municipalities in Denmark (NCT05480085) [17,18]. The purpose of this study was to assess the feasibility of the intervention, recruitment procedures, and data collection plan to identify elements requiring refinement prior to conducting the cluster RCT outlined in this protocol. Overall, stakeholders and participants in the feasibility trial expressed strong support for the study’s aims and found the intervention activities meaningful, while also highlighting areas that needed improvement. The feasibility study resulted in several refinements, which are reported in detail elsewhere [18]. First, the recruitment strategy was strengthened, providing clearer and more accessible information to parents and recognizing teachers as key gatekeepers in communication with families. Second, the intervention was adapted to enhance parental engagement and feasibility, including restructuring family meetings to be conducted at the class level rather than across the entire school, and providing additional guidance and support to facilitate local implementation. Third, data collection procedures were optimized, including simplifying and shortening the leisure time questionnaire, distributing it via SMS, and improving adherence to physical activity measurements through practical adjustments. Adjustments have been implemented and are reflected in the protocol for the Screen-Free Time With Friends cluster RCT presented here.

### Study Design and Participants

To evaluate the Screen-Free Time With Friends intervention, we conduct a cluster RCT involving children aged 9‐11 years from public schools across Denmark. The initial recruitment target was 18 schools, based on sample size calculations using our primary outcome: time spent with peers (in minutes). Schools are randomized by an independent statistician in a 1:1 ratio to either an intervention or a control group, balancing on the following criteria: (1) number of students in 3rd grade, (2) school-level parents’ ethnicity, (3) school-level parents’ education level, and (4) building coverage. A covariate-constrained randomization procedure is applied [19] to optimize the balance in school-level covariates between the intervention and control groups, thereby reducing the likelihood of substantial differences in child baseline characteristics. Data on school-level covariates are sourced from the Danish Ministry of Children and Education [20]. The statistician communicates the allocation results to the research team in two rounds, after the respective schools have completed their baseline assessments. Following randomization, after-school clubs and local community stakeholders affiliated with the schools in the intervention group are recruited to participate in the intervention. The respective school, after-school club, and local community will hereafter be referred to as an intervention school district. Intervention school districts implement the intervention program, consisting of five core components—(1) curricular activities, (2) two family meetings, (3) a brief exercise for parents, (4) an after-school club developmental program, and (5) two workshops in the local community—while school districts randomized to the control group continue with their usual practices.

### Recruitment

#### Schools

To recruit schools for the cluster RCT, school leaders are contacted via the municipality or directly by a member of the research team. Schools are eligible if they meet the following criteria: (1) the school must have an after-school or youth club (or similar) within walking distance and (2) the after-school club’s leader and staff must be willing to develop the club by participating in an after-school development program facilitated by Ungdomsringen. Ungdomsringen is a Danish nongovernmental organization that supports the development of youth clubs and educational environments through training, networking, and practice-based resources. No additional inclusion or exclusion criteria are applied, as the aim was to include a representative sample of children and parents from the participating 3rd-grade classes.

#### Children and Parents

In collaboration with a representative from the school, the research team recruits children and parents from 3rd grade at the participating schools. Based on the results from the feasibility trial [18], the strategy for informing and recruiting parents was carefully designed, incorporating multiple methods of communication and several reminders via Aula, which is an electronic communication platform at the schools, to encourage parents to enroll in the study. Initially, parents receive written information about the project along with a short video presentation of the project. In addition, the project is introduced to the children during school hours. Each child receives a keychain featuring the project logo, along with written information to take home and share with their parents. Parents are informed about this presentation via Aula. Lastly, parents are invited to an in-person information meeting at the school. At the information meeting, the project is comprehensively explained, and parents receive detailed written information along with the opportunity to ask questions.

#### After-School Club Staff

Following randomization, the contact information of the after-school club leaders in the intervention school districts is shared with Ungdomsringen. Ungdomsringen is responsible for the remaining practical planning of the after-school club development program, including the coordination of the 3 workshops with the leader and staff. The implementation of the program is planned with close consideration of each club’s schedule and preferences. Based on insights from the feasibility study, clubs with only a few staff members are merged with other clubs to optimize the workshop process and foster greater knowledge sharing.

#### Local Stakeholders

To support the recruitment of participants for the local workshops, a local ambassador is recruited in each of the 9 intervention school districts. The ambassador should represent the local community and possess knowledge of children’s leisure activities and opportunities in the area. The ambassador is invited to an initial meeting—either online or in person, depending on their preference—and receives informational materials, including a video outlining the aim and overall content of the 2 local workshops. The ambassador is responsible for recruiting 15‐18 local stakeholders who are engaged in or committed to promoting children’s leisure time activities in the local area (eg, representatives from the local sports club, church, or local politicians). The research team provides support as needed throughout the recruitment process.

### Ethical Considerations

The study was approved by the Research Ethics Committee at the University of Southern Denmark on June 15, 2022 (22/29625), and an updated version of the study protocol was approved on October 2, 2023. The study was presented to the Regional Research Ethics Committee, which determined that formal ethical approval was not required according to Danish legislation. Nevertheless, the research team sought an ethical review from the local institutional ethics committee at the University of Southern Denmark. The committee requested written parental consent for the physical activity measurements using accelerometers. For all measurements, including questionnaires, smartphone use monitoring, and measures of children’s social interaction, data are collected under the legal basis for research purposes in accordance with the Danish Data Protection Act (Section 10). Under this framework, individual consent is not required when data are used solely for research purposes, and appropriate safeguards are in place. To receive questionnaires, parents must register for the project, and participation in smartphone use monitoring requires parents to actively install an app themselves. For all measurements, parents are thoroughly informed about the nature and purpose of the data collection. Besides the information meeting, parents receive written information about the study and may formally withdraw their child from all assessments at any time. On the day of measurement, if a child indicates that they do not wish to participate, their decision is respected.

### Intervention Development

The Screen-Free Time With Friends intervention is the result of a comprehensive and systematic development process, guided by the Medical Research Council (MRC) framework for developing complex interventions [21] and inspired by the process outlined by Hawkins et al [22]. Consistent with the MRC framework, emphasis was placed on contextual relevance, stakeholder engagement, identification of key uncertainties, and iterative refinement of the program theory during the intervention development process. A wide range of stakeholders contributed to shaping the overall structure of the program—including researchers, practitioners (eg, schoolteachers and after-school club staff), end users (ie, children and parents), and organizations working with screen use and children’s leisure time. For example, an expert-generated systems map provided valuable insights into the multiple factors influencing children’s leisure activities and helped identify key leverage points for intervention, thereby informing the development of the intervention’s overall framework [21]. The final program protocols and materials were developed in close collaboration with national organizations, including Ungdomsringen, Just Human, a nonprofit foundation dedicated to promoting mental health and well-being among children and young people, and researchers with knowledge and experience in approaches inspired by Group Model Building (GMB) [23].

### The Screen-Free Time With Friends Intervention

#### Overview of the Intervention

**Figure 1.**
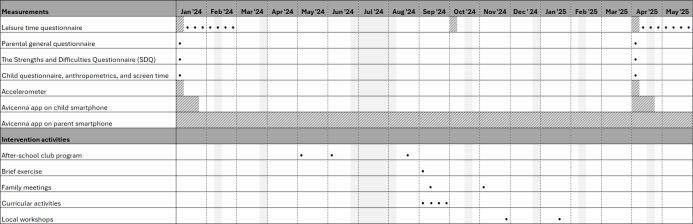
Example timeline showing the scheduling of data collection and intervention activities in a single-intervention school district. A dot represents one measurement or intervention activity. Bars with diagonal lines indicate measurement periods lasting 7 consecutive days per week (eg, accelerometer data collection). School holidays are represented by solid light gray bars.

The final intervention consists of five core components comprising (1) curricular activities targeting 9‐11-year-old children in each school class; (2) two family meetings targeting parents from each school class; (3) a brief exercise for parents, focusing on screen media dilemmas, included in the regular parental meeting at the start of 4th grade; (4) a developmental program targeting after-school club staff; and (5) two workshops in the local community targeting local stakeholders. Schools initiate and complete the intervention activities at different time points, depending on the timing of their allocation. Figure 1 illustrates an example of the intervention’s implementation in a single intervention school district.

#### Implementation Preparation and Support

Parents, schoolteachers, and after-school club staff in each intervention school district receive a video and written materials explaining the full intervention program prior to intervention start. A school representative is invited to an initial meeting with a member of the research team, where details about the family meetings and curricular activities are presented.

The school representative is responsible for ensuring that invitations to the family meetings are distributed to all relevant parents. They also receive information about the brief exercise for parents to conduct during the regular parental meeting at the start of 4th grade.

To support the implementation of the curricular activities, participating teachers are invited to a webinar introducing the materials. The webinar also provides an opportunity for teachers to ask questions related to both the material and the family meetings.

The after-school club leader is contacted by Ungdomsringen to coordinate the implementation of the after-school club development program.

The school representative is encouraged to recruit a local community representative (ie, a local ambassador) familiar with children’s leisure activities in the area. A video and written information describing the purpose of the local community workshops, along with an invitation and registration link, are sent to both the school and the local ambassador. They are encouraged to share this information broadly within the local community to recruit participants for the local workshops. The research team also supports the recruitment of both the local ambassador and local stakeholders.

All family meetings, local workshops, and after-school club workshops are facilitated by trained staff provided by the project.

#### Intervention Components

##### Intervention Component 1: Curricular Activities for Children

The curriculum activities include activities addressing screen media use and leisure time. The teaching material consists of videos, posters, workbooks, sheets, and reflection and dialogue tasks, which take place in groups or as a whole class. The program is structured into 4 modules comprising a total of 7 lessons (45 minutes/lesson), as outlined in Table 1. Teachers receive a printed teacher’s manual, a booklet outlining the theory and key concepts behind the material, and 5 posters to put up in the classroom. Teachers are encouraged to take breaks during the sessions to discuss with the children what they have read and learned while working through tasks in the workbooks. Additionally, teachers should use the provided videos and posters as tools for fostering dialogue during and after the lessons. Teachers are expected to implement the material from all modules but are welcome to spread it over more than 7 lessons. The material must be completed prior to the second family meeting—and no later than the autumn break in October 2024—as selected responses from the children are used as input for the meeting. The curricular activities aim to foster a shared understanding and vocabulary related to screen use and leisure habits within the class, and to lay the foundation for conversations between children and their parents on these matters.

**Table 1.** Description of the curricular activities targeting children aged 10‐11 years.

Module	Lesson	Title	Aims	Curricular activities
1	1‐2	Social communities and leisure time	The students develop an understanding of the importance of maintaining a good balance between different activities throughout the day. The students become able to reflect on their own leisure time activities and screen use, and understand that these factors affect their well-being.	A video that can be used to introduce the module. Posters depicting: (1) How participation in different communities and activities affects us and (2) the balance of activities in one’s daily life. Exercise “Swipe” (with movement): Questions are displayed on the smartboard and read aloud by the teacher. Children move either to the right (yes) or to the left (no). Responses are collected—to be used for the second family meeting. Exercises (workbook): Knowledge about social communities and how participation in different communities and activities affects us. Focus on the balance of activities in one’s daily life. Exercise “What would you prefer?” (worksheet): A checklist where students choose what they prefer from two statements. Responses are collected—to be used for the second family meeting.
2	3‐4	Digital and analog communication	The students gain an understanding of digital and analog communities and communication.	A video that can be used to introduce the module. Exercise (worksheets): Differences between digital and analog communication. Group work (3‐4 students): Reflection and dialogue about various digital communication situations/cases. Class discussion and development of “the class’s top 3 tips for think before you text.”
3	5‐6	Critical sense in the digital world	The students develop a certain degree of critical awareness and a conscious and reflective approach toward the digital environment. The students understand the importance of involving their parents in their digital lives.	A video that can be used to introduce the module. Posters depicting “The Critical Code”: “The Critical Code” consists of two points: (1) “pause and think” and (2) “involve an adult.” Exercises (workbook): Text and tasks that introduce “critical awareness in the digital world” and “The Critical Code.” Concepts such as algorithms and tech giants are introduced. Parents are described as superheroes who are ready to help—also in the digital world. Group work (3‐4 students): Reflection and dialogue about different cases, using “The Critical Code.” Can also be done collectively with the whole class.
4	7	Summary	The students reflect on what they have worked on and learned in the 3 previous modules.	Reflection on what students have worked on and learned, revisiting the 3 videos, posters, and key concepts from modules 1, 2, and 3. Exercise (worksheet): The worksheet is divided into 3 sections, each illustrating the 3 modules. Each section includes a small reflection exercise where students must draw/write what they have learned, worked on, or concluded in the 3 modules. The students are to take the worksheet home and go through it with their parents.

##### Intervention Component 2: Family Meetings for Parents

Within each class, children and parents are invited to two family meetings. The family meetings comprise a workshop for parents that combines education and dialogue between parents, while children engage in fun and interactive activities. The overall goal of these meetings is to support parents in fostering balanced and sustainable leisure habits, within the class community and at home, with particular attention to screen use. Building on empirical insights from the intervention development phase and inspired by theoretical perspectives such as social support theory [24], the meetings provide parents with a possibility to share experiences and foster dialogue, helping parents gain strategies and confidence to address digital challenges effectively. An essential aspect of the meetings is to foster collective decision-making on key issues relevant to the specific class, which could be (in)appropriate screen content, online gaming, or social media use. By aligning on these matters, the goal is to reduce social pressure, strengthen unity, and promote the development of more sustainable rules [25,26].

The first family meeting focuses on screen-related aspects within the family, while the second meeting addresses children’s screen use within the class. As part of the second meeting, data collected from the children during the curricular activities (Table 1) is presented to the parents to ground the discussions in the children’s real-life experiences and perspectives. Evidence-based knowledge is shared through slides referencing research and recent reports as well as videos featuring expert statements. In addition, handouts are provided to parents, including practical guidance on how to install parental controls on a child’s smartphone and how to initiate conversations about screen use within the family. Workshops are led by a trained facilitator provided by the project, and all materials are freely available online following the meeting.

Activities for the children are conducted by staff from the after-school club or sport science students from the University of Southern Denmark, coordinated by the research team in collaboration with the school, based on their preferences. These activities, along with a concluding shared dinner, are included as part of the intervention to support and encourage parent participation by making attendance as convenient as possible.

##### Intervention Component 3: Brief Exercise for Parents

In Denmark, a class-wide parent-teacher meeting is held at the beginning of each school year. During this meeting in 4th grade, parents take part in a brief teacher-led exercise focusing on screen media dilemmas. The primary goal is to raise awareness about the project and encourage parents to participate in the upcoming family meetings.

##### Intervention Component 4: Development Program for After-School Club Staff

The development program consists of 3 workshops conducted within the after-school club setting. The program is developed in collaboration with Ungdomsringen, which facilitates the program.

The development program is inspired by principles of action learning [27], applied in a condensed, iterative format referred to as a “mini action learning cycle.” This means that the after-school club staff are expected to develop their own practice based on specific, real-life challenges and dilemmas they have experienced. In this way, the issues addressed during the process are directly relevant to their everyday practice, supporting ownership of the change process.

During the workshops, after-school club staff are supported in investigating identified challenges and preparing small actions to be implemented in their everyday practice. In the following session, their experiences and observations become the basis for systematic reflection, knowledge sharing, and the development of new insights—insights that can inform further actions. As part of the program, the after-school club staff are introduced to knowledge, tools, and methods that enable them to continue developing their practice—even after the formal conclusion of the development program. The overall aim of the program is to increase participation in the after-school club and reduce dropout rates from 3rd to 4th grade. To achieve this, the program, for example, seeks to foster a shared understanding of current practices across key themes, and strengthen empowerment and a sense of unity among the club’s staff.

##### Intervention Component 5: Community Workshops for Local Stakeholders

The last intervention component comprises two 3-hour workshops in the local community. A local ambassador and 15‐18 local stakeholders are recruited from the local community, representing parents, schoolteachers, after-school club personnel, local politicians, and others relevant to children’s leisure time (representatives from, for example, local sports clubs, library, and church). With support from the research team, the local ambassador is responsible for planning the two workshops, which are led by trained facilitators. The workshops are based on the GMB methodology, aiming to foster a shared understanding of the problem by collaboratively developing a system dynamics model grounded in diverse stakeholder perspectives [28]. GMB has been shown to enhance problem understanding, strengthen stakeholder engagement and confidence in systems thinking, and foster consensus for action [23,29,30]. Based on experiences from the feasibility study and the fact that the local workshops are part of a multicomponent intervention, the decision was made to limit the workshop component to two 3-hour sessions.

In the first workshop, stakeholders identify key factors and collaboratively develop a visual representation of the system, illustrating various factors that influence children’s face-to-face interactions with peers during leisure time in the local area—and how these factors are interconnected. The purpose of the system map is to help stakeholders understand the system’s dynamics and support system-level change [31]. During the second workshop, and based on the system map, stakeholders identify existing actions and prioritize key leverage points for intervention. Local actions are then outlined, and action groups are formed based on individuals’ interest, motivation, and capacity to drive change within the specific area. The overall goal of the workshops is to increase opportunities for children to engage in face-to-face leisure activities with peers. This is supported by building shared awareness and understanding of the challenge that children spend more time on screens and less together in person, and fostering empowerment, a sense of unity, and supportive social networks to take collective action.

### Effectiveness Evaluation

#### Overview of Outcome Measures

All outcome measurements are conducted at all 18 schools at baseline (T0) and again after 13‐15 months. In addition, the leisure time activity questionnaire—which includes the primary outcome of time spent face-to-face with peers—is administered at T0 + [6‐10 months]. An overview of the measurements is provided in Table 2. Below, outcomes related to the primary target groups (ie, children and their parents) are detailed.

**Table 2.** Overview of outcomes, measurement methods, time points, and information on data source and target (ie, the person or group the outcome concerns). The table presents the outcomes assessed at each time point, along with the corresponding measurement methods. For each outcome, the table specifies who the outcome concerns (target) and how data were collected (data source). For example, when parents report on their child’s screen use, the child is the target, and the data source is specified as parent-report. For subjective measures, the respondent is indicated; for digital measures, the source is specified (eg, app-based or device-based).

Outcome	Target	Measurement	Data source	Time points
Total time spent face-to-face with peers, time spent face-to-face with peers in different domains, and solitary screen use	Child	Leisure time activity questionnaire	Parent-report	T0, T0+(6‐10 months), and T0+(13‐15 months)
Total screen media use and screen media addiction	Child	General parental questionnaire	Parent-report	T0 and T0+(13‐15 months)
Smartphone addiction	Parent	General parental questionnaire	Parent-report	T0 and T0+(13‐15 months)
Family dynamics related to screen use	Family	General parental questionnaire	Parent-report	T0 and T0+(13‐15 months)
Physical activity and sleep	Child	Axivity AX3 triaxial accelerometer	Device-based	T0 and T0+(13‐15 months)
Smartphone time	Parent	Avicenna app	App-based	T0 and T0+(13‐15 months)
Smartphone time	Child	Built-in screen time tracking features (iOS/Android)	Device-based (retrieved by research team)	T0 and T0+(13‐15 months)
Well-being	Child	The Strengths and Difficulties Questionnaire	Teacher/pedagogue-report	T0 and T0+(13‐15 months)
Social relations and class cohesion	School class	Child questionnaire	Child-report (during school hours)	T0 and T0+(13‐15 months)

#### Children’s Time Spent Face-to-Face With Peers

To assess the primary outcome, children’s face-to-face time with peers during leisure, parents receive an SMS-based leisure time activity questionnaire about their child’s leisure activities for 7 consecutive days, followed by once-weekly messages for 7 weeks, during both the baseline and follow-up periods. Midway, they receive the same questionnaire for 7 continuous days. The questions cover the amount of time children spend in after-school clubs, organized leisure activities (eg, sports or crafts), and unorganized time with peers such as playdates. Total face-to-face interaction time with friends during leisure is calculated as the sum of these 3 categories.

#### Children’s Solitary Screen Use

As part of the SMS-based leisure time activity questionnaire, parents are asked to report on children’s time spent using screen media devices (including smartphone, tablet, television, and computer) alone. Screen time is defined as “alone” when the child is using a screen without others in the same room being actively involved in the activity. For example, online interaction with others still qualifies as alone time, whereas shared use—such as interacting together on a tablet—does not.

#### Children’s Physical Activity and Sleep

Children’s physical activity is assessed using Axivity AX3 triaxial accelerometers [32] worn at the thigh using two adhesive patches 24 hours/day for 7 consecutive days at baseline and follow-up [33]. Time spent in different intensity domains of physical activity, physical activity types, and sleep metrics is determined using the appropriate algorithms, such as those developed by Brønd et al [34]. Children with at least 3 valid weekdays and 1 valid weekend day will be considered compliant with the monitoring protocol. Preparation of the accelerometers and download of data will be conducted using the OmGui software [35].

#### Child Smartphone Time, Total Screen Media Use, and Addiction

Parents are asked to install an app on their child’s smartphone if the child owns one [36]. The app objectively monitors smartphone use over 3 weeks at both baseline and follow-up by logging screen activation and deactivation events. In addition, the research team retrieves data on children’s smartphone time from the past week as part of the assessment battery conducted during school hours at both baseline and follow-up.

Using a modified version of the SCREENS-Q [37], children’s overall screen media use is parent-reported at baseline and follow-up. Moreover, parents report their child’s screen media addiction using the Problematic Media Use Measure—Short Form (PMUM-SF) [38]. The PMUM-SF is a validated 9-item parent-report scale designed to capture addictive-like patterns of screen media use in children aged 4-11 years, based on criteria reflecting impaired control, preoccupation, withdrawal, and family conflict [38].

#### Child Well-Being

Child well-being is assessed using the Strengths and Difficulties Questionnaire for teachers, consisting of 25 items focusing on emotional symptoms, conduct problems, hyperactivity/inattention, peer relationship problems, and prosocial behavior at baseline and follow-up [39,40]. In addition, as part of the child questionnaire, children are asked to complete Cantril’s Ladder [41], a widely used single-item measure of global life satisfaction.

#### School Class, Social Relations, and Cohesion

To assess peer relationships and social structures within the school class, children are asked—as a part of a child questionnaire—to indicate which classmates they spend the most time with during recess and leisure activities. These data are used to construct sociograms, enabling analysis of social networks, including indicators such as centrality, mutual connections, and potential social isolation [42]. In addition, children will report on perceived class cohesion.

#### Parent Smartphone Time and Addiction

As for the children, parents are asked to download an app [36] to their own smartphone to track smartphone use throughout the intervention period. Smartphone addiction is self-reported using the Smartphone Addiction Scale—Short Version (SAS-SV) at baseline and follow-up [43]. The SAS-SV is a validated 10-item questionnaire designed to assess symptoms of problematic smartphone use, including daily-life disturbance, withdrawal, tolerance, and preoccupation.

#### Family Dynamics Related to Screen Media Use

As a part of the general parental questionnaire, parents are asked to report on the frequency of conflicts in the family related to screen media use.

### Process Evaluation

A comprehensive process evaluation is conducted, guided by the framework of Moore et al [44] and the program theory. The process evaluation will explore three key domains—(1) implementation, (2) mechanisms of change, and (3) context—as recommended by Moore et al [44]. The primary purpose of the process evaluation is to provide an understanding of the implementation of the intervention and relevant contextual factors. In addition, the process evaluation will enable exploration of potential mechanisms through which the intervention may influence outcomes.

All intervention components follow standardized manuals and predefined structures to support consistency across settings. Fidelity is assessed using structured facilitator checklists completed after each session (ie, facilitators of family meetings, local workshops, the after-school club development program, and teachers implementing the curricular activities), documenting whether predefined components were delivered and to what extent they were implemented as intended. Information about reach (extent of participation) and any adaptations or deviations from the protocol are also recorded by the facilitator. In addition, participants are asked to complete questionnaires immediately after participating in each respective intervention component, except for the curricular activities and the brief exercise. This together will enable us to understand how the intervention was implemented and received across different settings.

To explore mechanisms of change, we gather information about potential mechanisms identified in the program theory, including, but not limited to, perceived social support, collective agency, and actions. This information is obtained at multiple time points, depending on the component: before, immediately after, and at follow-up for the local workshops and the after-school club program; and immediately after and at follow-up for the family meetings and the educational material. To obtain a more nuanced understanding, semistructured interviews are also conducted in some cases. For example, to capture a more complete picture of actions initiated as a result of the local workshops and after-school club program, we plan to interview at least one participant per local workshop or after-school club.

To gain an understanding of the different contexts in which the intervention is implemented, we explore contextual factors (eg, organizational structures, local resources, and existing practices) that may influence implementation and outcomes. These data are collected through interviews and questionnaires targeting key informants (ie, representatives of the local community, school, and after-school club).

Based on insights from the feasibility study and in alignment with the recommendations by Moore et al [44], we recognize the need for a flexible, multimethod approach in the collection of qualitative data. While interviews are the preferred method for exploring participants’ experiences in depth, open-ended questionnaires are used when participants decline to take part in interviews or when scheduling interviews is not feasible. This approach allows participants to provide written reflections in their own words and helps ensure that perspectives from a broader range of participants are captured in the process evaluation.

### Data Management

Questionnaire data are collected using SurveyXact (Ramboll), except for the data from children (ie, child questionnaire), which is obtained through the system Klassetrivsel (Skolevisioner ApS). This platform enables the collection of information for assessing peer relationships and social structures within the school class and offers the option of having questions read aloud. All data—including questionnaire responses, audio recordings and transcriptions from interviews, and data from objective measurements—will be extracted and securely stored on servers hosted by the University of Southern Denmark. Data are stored in their raw form.

### Statistical Methods and Analysis

#### Sample Size Calculation

We anticipated a total of 1080 eligible 3rd-grade children, assuming an average of 2.5 classes per school and 24 students per class in each of the 18 schools. With an expected participation rate of approximately 80%, we aimed to enroll 864 children at baseline. Accounting for an estimated 15% dropout or missing data rate, we projected an average of 41 children per school (cluster) in the final analytical sample, resulting in a total sample size of approximately 738 children. Drawing on a previous Danish school-based study conducted by our research group [45], we assumed a coefficient of variation in cluster size of 0.24 and an intracluster correlation coefficient of 0.07. The intraclass correlation coefficient estimate was based on internal calculations from that study using a related behavioral outcome (physical activity), as direct estimates for the present primary outcome were not available. Additionally, based on findings from the feasibility trial, we estimated a correlation of 0.67 between baseline and follow-up measurements, and a standard deviation of 38 minutes per day in both the intervention and control groups. To control for type I error due to the small number of clusters, we lowered the α level to .025 in the power calculation. This adjustment was made in anticipation of applying the Satterthwaite correction for mixed linear models, as recommended for small-sample cluster trials [46]. Under these assumptions, the study is powered at 80% to detect a statistically significant mean difference of 13.5 minutes per day or more between the intervention and control groups at follow-up. A difference of 13.5 minutes per day corresponds to approximately 1.6 additional hours of peer interaction per week, roughly equivalent to one extra weekly after-school playdate or an extra organized extracurricular activity. Such changes may be meaningful, as previous research has shown that engaging in social leisure activities, including meeting friends at least once a week, is positively associated with adolescents’ well-being and perceived peer support [47]. Moreover, social connection has been consistently associated with better physical and mental health [48]. This interpretation is further supported by Danish data indicating that a substantial proportion of 11-year-olds have relatively low levels of face-to-face interaction with peers, with approximately 50% reporting that they meet friends once a week or less, or not at all [9]. Together, this supports that the chosen effect size may represent a meaningful change in children’s everyday social interaction.

#### Baseline Characteristics

Baseline characteristics will be presented descriptively for the intervention and control groups using appropriate summary statistics. In addition, the balance for the school-level covariates included in the constrained randomization procedure (number of students in 3rd grade, parental ethnicity, parental education level, and building coverage) will be reported.

To assess sample representativeness, baseline characteristics will be compared between children with at least one participating parent and those without participating parents using data available for the majority of children. These comparisons will include selected child-reported measures (eg, indicators of well-being and leisure activities), as well as teacher-reported Strengths and Difficulties Questionnaire scores.

#### Effectiveness Evaluation

##### Primary Outcome

The primary outcome is parent-reported total face-to-face interaction time with friends during leisure, assessed using the SMS-based leisure time questionnaire. The primary end point is the between-group difference from baseline to 13‐15 months follow-up.

##### Secondary Outcomes

Secondary outcomes include children’s time spent face-to-face with peers categorized into distinct time domains (after-school club, organized activities, and unstructured time with peers) and solitary screen use during leisure time assessed using the SMS-based leisure time questionnaire. Secondary end points are between-group differences from baseline to 13‐15 months follow-up.

In the clinical trial registration, 5 secondary outcomes were prespecified, including physical activity. However, due to time and resource constraints, physical activity will not be included in the primary publication and will instead be reported separately. Consequently, this outcome will be treated as exploratory. The remaining 4 secondary outcomes will be included in the primary publication alongside the primary outcome and analyzed within a confirmatory framework with appropriate adjustment for multiple testing.

##### Primary Analysis

Mixed effects regression models will be used to estimate between-group differences from baseline to follow-up in time spent face-to-face with peers. Models will be adjusted for weekday and include school, participant ID, and measurement round as random effects to account for clustering and repeated measurements. Imbalanced covariates will be included as fixed effects if relevant. The Satterthwaite correction will be applied to account for the small number of clusters, as recommended [46]. All analyses will follow the intention-to-treat principle.

##### Secondary Analyses

Analyses of secondary outcomes will follow the same modeling approach as the primary analysis. Adjustment for multiple testing will be applied using the Holm step-down procedure to control the familywise error rate at a 2-sided significance level of α=.05. Adjusted *P* values will be reported for the secondary end points, and findings will be reported alongside the primary outcome to aid interpretation of the results.

For the additional outcomes (eg, physical activity and well-being), models will include school and participant ID as random effects. No adjustment for multiple testing will be applied for these outcomes, and findings will be interpreted as exploratory.

##### Missing Data

For the primary and secondary outcomes, participants with at least one valid day of SMS-based data will be included in the analysis. Mixed effects models allow inclusion of participants with varying numbers of observed days, thereby retaining available information and maximizing statistical power.

##### Sensitivity Analyses

Given that the primary outcome is ordinal, the primary analysis will be repeated using mixed ordinal logistic regression with the outcome on its original scale.

For all analyses, hypothesis tests will be 2-sided with a significance level of α=.05. Statistical analyses will be conducted using Stata or R (R Foundation for Statistical Computing).

### Process Evaluation

Process evaluation data include both quantitative and qualitative sources. Quantitative process data (eg, on fidelity, reach, and dose delivered) will primarily be analyzed descriptively. Qualitative data will be analyzed thematically to identify patterns in participants’ experiences, contextual influences, and potential unanticipated and anticipated change mechanisms. Although the trial is not powered for formal mediation analyses, exploratory analyses may be conducted to examine whether selected process indicators related to implementation, mechanisms of change, or context are associated with the observed intervention outcomes. Statistics will be conducted using Stata or R (R Foundation for Statistical Computing), and qualitative data will be managed and coded in NVivo (Lumivero).

## Results

This cluster RCT was funded in July 2020 as part of a larger project that also included a formative qualitative study, an intervention development process, and a feasibility study [17,18]. For this cluster RCT, schools were recruited from July 2023 to January 2024, and children and parents were recruited from November 2023 to April 2024. At baseline, 685 children were enrolled at the participating schools (Table 3). During the study period, 50 transferred to nonparticipating schools, while 32 were newly enrolled at participating schools, resulting in 667 children being enrolled at the participating schools at follow-up. In addition, 2 children moved between schools within the study. All children participated in most of the measurements unless they were actively withdrawn by their parents or absent from school on the day the measurements were conducted. Table 3 provides further details about the participant groups, including the number of children with consent to participate in the physical activity measurements, as well as the number of children with at least one parent who voluntarily enrolled to complete questionnaires and install the Avicenna app.

**Figure 2.**
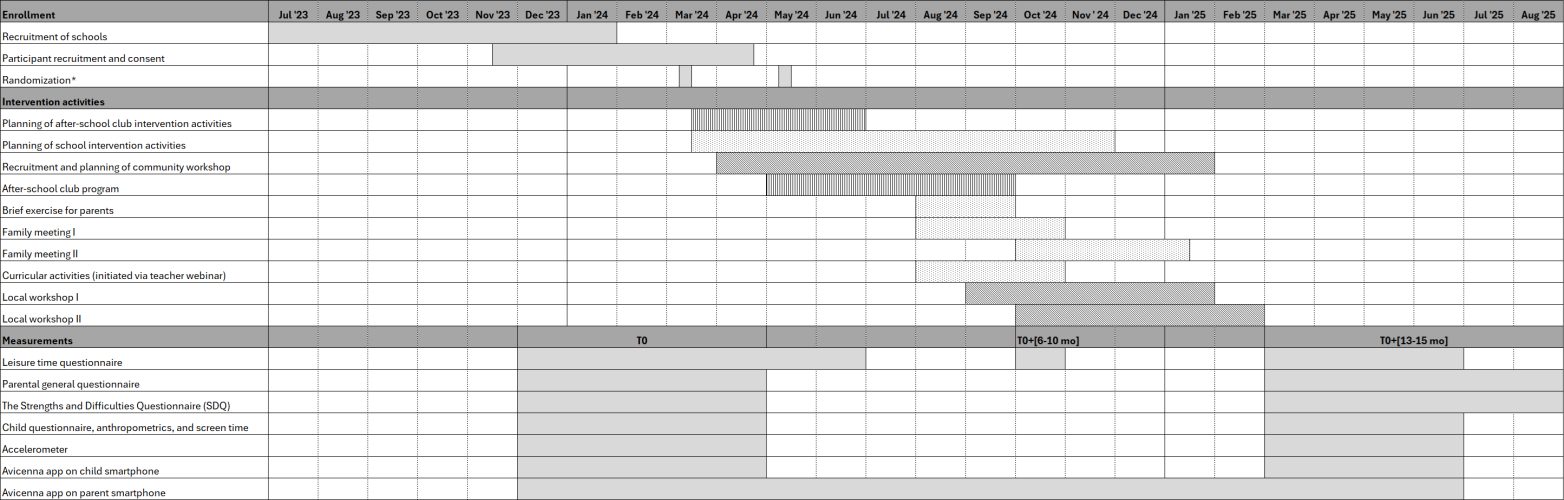
Overview of recruitment, intervention activities, and measurements. Patterns indicate the settings in which intervention activities are planned or implemented: vertical lines = after-school club; dots = school; diagonal lines = local community. *The randomization of schools was conducted in March 2024; however, the allocation results were communicated to the research team in two rounds: round 1 (7 schools) and round 2 (11 schools).

Baseline data were collected from December 2023 to June 2024. Schools were randomized in March 2024, with allocation results communicated to the research team in two rounds (round 1: 7 schools; round 2: 11 schools). The scheduling and implementation of the intervention activities ran from March to May 2024, when the children were in 3rd grade (ages 9‐10 years), to March 2025, when they completed 4th grade (ages 10‐11 years). The exact timing of intervention activities varied by school district, as scheduling was coordinated with schools, after-school clubs, and local communities to ensure feasibility. Follow-up data collection took place from March 2025 to August 2025. A study timeline is presented in Figure 2 [49]. Interim data checks have been performed to ensure data quality; however, no formal statistical analyses of intervention effects have been conducted (as of March 2026).

**Table 3.** Participation status of children and parents at baseline and follow-up.

Participating children	Baseline, n (%)	Follow-up, n (%)
Children enrolled at the participating schools	685 (100)	667 (100)
Children *not* withdrawn by parents	675 (99)	642 (96)
Children with consent to participate in accelerometer measurements	366 (53)	372 (56)
Children with ≥1 parent enrolled in questionnaire	343 (50)	351 (53)

As of March 2026, no study results are currently available. Findings on the primary and secondary outcomes and on the implementation of the intervention are expected to be published by the end of 2026. Results will be disseminated regardless of outcome through peer-reviewed scientific journals and presentations at relevant scientific conferences. There are no publication restrictions, and the investigators retain full authority over the dissemination of findings.

## Discussion

### A Multicomponent Approach to Children’s Leisure Behaviors

This paper describes the study protocol for the Screen-Free Time With Friends cluster RCT. The study aims to evaluate the effectiveness of the pragmatic intervention program on a range of outcomes, including—but not limited to—time spent face-to-face with friends, solitary screen time, well-being, classroom social relationships and cohesion, and physical activity among children aged 9‐11 years.

As children’s leisure time behaviors are shaped by a variety of factors [50-56], the Screen-Free Time With Friends intervention is developed with a systemic perspective combining family meetings, curricular activities, development of the after-school club, and an opportunity for local stakeholders to address the diverse aspects influencing children’s face-to-face interactions and screen use. While the intensity of each individual component may be insufficient to generate system-level change on its own, the components are intended to act as catalysts for dialogue, engagement, and local action when implemented in combination.

In recent years, both public discourse and scientific literature have increasingly highlighted the importance of parental dialogue and consensus—such as the development of shared principles for children’s screen use—as a potential strategy to address the negative aspects associated with children’s screen use [25,26]. However, despite growing recognition of the need for coordinated parental action, few studies have provided concrete, structured approaches or evaluated their effectiveness and feasibility in school settings. The family component of the Screen-Free Time With Friends intervention offers a structured and scalable approach to this challenge. Regardless of the findings from the effectiveness evaluation, the accompanying process evaluation will provide insights into how this component functions in practice and how parental engagement in managing children’s screen use can be supported.

In Denmark, structured after-school clubs play a central role in children’s leisure time [57] and provide inclusive spaces for peer socialization. Most public schools in Denmark transition children from one type of after-school club to another between grades 3 and 4 (ages 9‐11 years). While 94% of children are enrolled in an after-school club in grade 0, participation drops to just 50% by grade 4, with the largest decline occurring between grades 3 and 4 [57,58]. As a result, more children begin spending their afternoons alone at home, which has been found to be associated with higher screen use [13]. Increased participation in after-school clubs during the transition from grade 3 to 4 may help ensure that more children spend their leisure time in face-to-face interaction with peers rather than alone at home. The after-school club component of the Screen-Free Time With Friends intervention provides a structured approach that may help reduce dropout between 3rd and 4th grade and increase participation on a daily basis. However, to make this approach scalable, additional resources are needed to fund facilitator salaries and to provide time and support for staff to further develop their clubs—also beyond what is included in the 3 sessions.

The use of methods related to GMB at the local level has increasingly been applied to address complex public health challenges [23]. In this study, the local workshops are informed by the GMB methodology and build on insights from previous studies, including the Screen-Free Time With Friends feasibility trial [17,18]. While the intervention includes only two workshops of 3 hours each—less time than typically reported in comparable GMB-based interventions [23]—findings from the feasibility trial suggest that even this relatively limited effort initiated concrete local actions in communities that completed both workshops [18]. This may indicate that the workshops can act as a catalyst for local engagement and action.

Civic engagement and volunteer participation are under pressure in Danish society, which may challenge workshop attendance and limit time and resources for follow-up activities. Our feasibility trial also indicated that it was not possible to implement the workshops successfully in all settings. Conducting this larger-scale trial will allow us to examine whether certain types of local contexts are less conducive to this approach. Moreover, the study will provide insights into key conditions for successful implementation, including recruitment and participation, the role of the local ambassador, and practical considerations related to facilitating community engagement. These findings will help inform future implementation and potential scale-up if the intervention proves effective.

### Anticipated Limitations

During the feasibility trial, recruiting parents to sign up for the study proved challenging [18], which is critical for evaluating the intervention’s effectiveness on outcomes that rely on data collected from enrolled parents. To address the challenge of enrolling parents in the study, the cluster RCT implemented several strategies based on recommendations from the feasibility trial. Despite these efforts, only around 50% of the children included in the study had at least one parent enrolled in the questionnaire component; therefore, the primary outcome is based on a subset of the study population. Participating families may differ systematically from nonparticipating families, including with respect to socioeconomic position and ethnic background. This may introduce selection bias, reduce the representativeness of the analytic sample, and limit the external validity of the findings. To explore this, baseline characteristics will be compared between children with at least one participating parent and those without participating parents using data available for the majority of children. This will provide an indication of the extent to which participating and nonparticipating children and families differ, which will be explicitly considered in the interpretation of the study findings, particularly with respect to the generalizability of the results.

It should be noted that the SMS-based questionnaire has not been formally validated. However, it was developed for this study and tested in a feasibility study, which informed adjustments to improve clarity and feasibility. Clear definitions were provided for each question, and the questionnaire was explained during oral information meetings with parents to support consistent understanding. While formal validation may strengthen the measurement validity, this approach was considered appropriate given the focus on simple, factual questions about same-day behaviors, which are less cognitively demanding to report and may be less susceptible to recall bias compared to more complex or retrospective measures.

Another potential limitation of the study is the relatively short duration of the intervention period. A short intervention may limit the potential to produce broader systemic changes, which may require longer-term engagement or repeated interventions. However, the intervention targets key leverage points, including parental and community engagement, which may, over time, facilitate meaningful changes in children’s opportunities for face-to-face interaction. Thus, even if measurable effects are not observed within the timeframe of the cluster RCT, longer-term follow-up may be warranted to assess whether changes emerge over time.

Importantly, the process evaluation will, in addition to examining the implementation of the intervention, allow for a deeper understanding of whether the intervention initiates change that—although maybe not yet reflected in the primary outcomes—may lead to longer-term impacts.

### Implications

This cluster RCT will provide evidence on the effectiveness and implementation of the Screen-Free Time With Friends intervention. Findings may support stakeholders such as municipalities, schools, after-school programs, community actors, and parents in creating environments that promote children’s face-to-face interaction and reduce screen use. The results may also inform future initiatives, policy development, and further research on feasible, real-world interventions that foster meaningful and enriching leisure experiences for children.

## Abbreviations

GMB: Group Model Building
MRC: Medical Research Council
PMUM-SF: Problematic Media Use Measure—Short Form
RCT: randomized controlled trial
SAS-SV: Smartphone Addiction Scale—Short Version
SPIRIT: Standard Protocol Items: Recommendations for Interventional Trials
